# Asynchronous mHealth Interventions in Rheumatoid Arthritis: Systematic Scoping Review

**DOI:** 10.2196/19260

**Published:** 2020-11-05

**Authors:** Bart F Seppen, Pim den Boer, Jimmy Wiegel, Marieke M ter Wee, Marike van der Leeden, Ralph de Vries, Martin van der Esch, Wouter H Bos

**Affiliations:** 1 Amsterdam Rheumatology and Immunology Center Reade Amsterdam Netherlands; 2 Department of Rheumatology VU Medical Center Amsterdam UMC Amsterdam Netherlands; 3 Department of Epidemiology and Biostatistics, Amsterdam Public Health Vrije Universiteit Amsterdam Amsterdam UMC Amsterdam Netherlands; 4 Department of Rehabilitation Medicine VU Medical Center Amsterdam UMC Amsterdam Netherlands; 5 Medical Library Vrije Universiteit Amsterdam Amsterdam Netherlands

**Keywords:** mobile health, eHealth, digital health, telehealth, telerheumatology, mHealth, web app, smartphone app, activity tracker, rheumatoid arthritis, rheumatology, review, telemonitoring

## Abstract

**Background:**

Mobile devices such as smartphones and tablets have surged in popularity in recent years, generating numerous possibilities for their use in health care as mobile health (mHealth) tools. One advantage of mHealth is that it can be provided asynchronously, signifying that health care providers and patients are not communicating in real time. The integration of asynchronous mHealth into daily clinical practice might therefore help to make health care more efficient for patients with rheumatoid arthritis (RA). The benefits have been reviewed in various medical conditions, such as diabetes and asthma, with promising results. However, to date, it is unclear what evidence exists for the use of asynchronous mHealth in the field of RA.

**Objective:**

The objective of this study was to map the different asynchronous mHealth interventions tested in clinical trials in patients with RA and to summarize the effects of the interventions.

**Methods:**

A systematic search of Pubmed, Scopus, Cochrane, and PsycINFO was performed following the Preferred Reporting Items for Systematic Reviews and Meta-Analysis (PRISMA) guidelines. Studies were initially screened and later assessed by two independent researchers. Disagreements on inclusion or exclusion of studies were resolved by discussion.

**Results:**

The literature search yielded 1752 abstracts. After deduplication and screening, 10 controlled intervention studies were included. All studies were assessed to be at risk for bias in at least one domain of the Cochrane risk-of-bias tool. In the 10 selected studies, 4 different types of mHealth interventions were used: SMS reminders (to increase medication adherence or physical activity; n=3), web apps (for disease monitoring and/or to provide medical information; n=5), smartphone apps (for disease monitoring; n=1), and pedometers (to increase and track steps; n=1). Measured outcomes varied widely between studies; improvements were seen in terms of medication compliance (SMS reminders), reaching rapid remission (web app), various domains of physical activity (pedometer, SMS reminders, and web apps), patient-physician interaction (web apps), and self-efficacy (smartphone app).

**Conclusions:**

SMS reminders, web apps, smartphone apps, and pedometers have been evaluated in intervention studies in patients with RA. These interventions have been used to monitor patients or to support them in their health behavior. The use of asynchronous mHealth led to desirable outcomes in nearly all studies. However, since all studies were at risk of bias and methods used were very heterogeneous, high-quality research is warranted to corroborate these promising results.

## Introduction

### Background

An increase in the prevalence of rheumatoid arthritis (RA), as well as a general shortage of rheumatologists to treat patients with RA, has been described [1,2]. In combination with rising health care costs, this makes structural changes in the Dutch health care system seem inevitable [3]. The challenge set is difficult, as solutions for the increasing health care costs, the rising prevalence of RA, and the subsequent demand on our outpatient clinics will need to follow the treat-to-target guidelines [4]. Integrating mobile health (mHealth) into daily clinical practice may help overcome the challenges the Dutch health care system is facing in the care of patients with RA and other chronic diseases [5-7]. Especially for patients with RA, the anticipated benefits of mHealth use should be evaluated, as adaption of mHealth might be challenging for older patients with RA or patients impeded by hurting joints [8,9].

The World Health Organization defines mHealth as “medical and public health practice supported by mobile devices such as mobile phones, tablets (…)” [7]. Apps for gait analysis, activity tracking, and video consultations and devices for handgrip strength monitoring have been developed and tested in patients with RA [10-13]. Two types of mHealth interventions can be distinguished: synchronous interventions, such as tele- and video consultation (where health care provider and patient are in direct real-time contact), and asynchronous interventions (no direct real-time contact), such as electronic consultations and remote disease activity monitoring through web or smartphone apps [14,15]. Asynchronous mHealth interventions have not received the same degree of attention as synchronous mHealth in RA [16,17], despite the anticipated benefits such as shorter wait times, lower health care usage, and consultations tailored to need [18-21]. So far, it remains unclear what evidence exists for the use of asynchronous mHealth interventions in patients with RA.

### Objective

The objective of this scoping review was to map the different asynchronous mHealth interventions tested in clinical trials in patients with RA and to summarize the effects of the interventions. Ultimately, this should help to identify promising implementations and future research opportunities.

## Methods

### Study Design

We conducted a scoping review of the literature. This type of review is suitable to map the available evidence in new and developing fields. The value of scoping reviews to evidence-based health care and practice lies in the examination of a broader area to identify gaps in the research knowledge base, clarify key concepts, and report on the types of evidence that address and inform practice in the field [22].

### Search Methods

A review protocol was developed based on the Preferred Reporting Items for Systematic Reviews and Meta-Analysis (PRISMA) statement [23]. Consequently, a comprehensive search of the bibliographic databases PubMed, Embase.com, Ebsco/PsycINFO, Wiley/Cochrane Library, and Scopus was performed by a medical librarian. Databases were searched from inception to November 20, 2019. The following terms were used as index terms or free-text words (including synonyms and closely related words): smartphones, internet, eHealth, mHealth, wearable, apps, rheumatoid arthritis, and tele-rheumatology.

The search was performed without date, language, or publication status restrictions. Duplicate studies were excluded. The full search strategies for all databases can be found in Multimedia Appendix 1.

### Inclusion and Exclusion Criteria

The following inclusion criteria were used: (1) the study population comprised only patients with RA, or the majority of the study population consisted of patients with RA, for whom the data were reported separately, (2) the study evaluated an asynchronous mHealth intervention (ie, the health care provider and patient were not in direct synchronous contact), (3) the study type was randomized controlled trial (RCT), randomized controlled crossover trial, or quasi-experimental clinical trial, (4) the study reported outcomes in relation to the mHealth intervention, and (5) the study was published in English as a full-length paper and an original report.

Studies that reported only qualitative outcomes (eg, from focus groups, semistructured interviews, etc) were excluded from the review. References of retrieved studies were screened for additional relevant studies. Interventions that used a web app were taken into account, as these are easily accessible through mobile devices (smartphone and/or tablet) and therefore regarded as mHealth in this review.

### Selection of Studies

Initially, the title and abstract were screened independently for eligibility criteria and blinded to each other with the online tool Rayyan [24]. Full-text papers were retrieved for all abstracts that met the inclusion criteria. Disagreements on the inclusion or exclusion of studies were resolved by discussion.

### Data Extraction and Categorization

Data were extracted by one reviewer using a standardized template and verified by a second reviewer. The following data were extracted from each included study: the year of the study, the number of participants, patient characteristics, type of study, type of intervention, duration of study and follow-up, outcome measures, univariate outcomes, and statistical significance. Disagreements or discrepancies in data extraction were resolved by discussion.

### Quality Assessment of the mHealth Intervention Studies

The quality of each study was independently evaluated using the Cochrane Collaboration’s tool—a 7-domain tool for assessing risk of bias in which study characteristics are classified as having high, low, or unclear risk of bias [25]. For each study, all items were rated by the same reviewers and substantiated in a Microsoft Excel file (version 2013; Microsoft Corporation). The results were compared and disagreements in results were resolved by discussion and, when necessary, by consultation with a third reviewer.

## Results

### Study Selection and Inclusion

**Figure 1:**
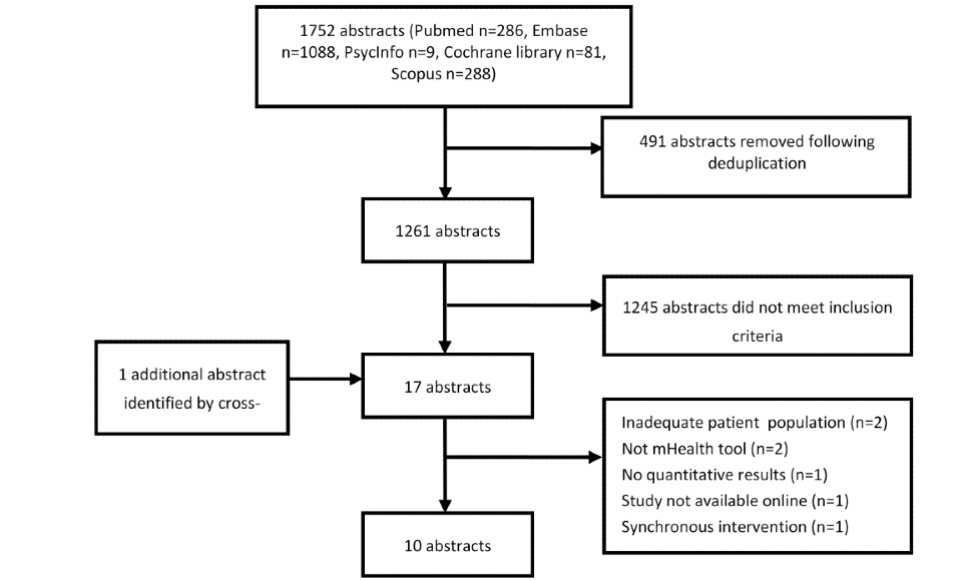
Study selection. mHealth: mobile health.

The search yielded 1752 abstracts (Pubmed: n=286; Embase: n=1088; PsycInfo: n=9; Cochrane library: n=81; and Scopus: n=288). One additional abstract was added after cross-referencing. After deduplication, 1261 abstracts remained, of which 1245 did not meet the inclusion criteria. The main reasons to exclude abstracts were that they were not mHealth studies, the population was not patients with RA, they were conference abstracts, or they were the wrong study type (ie, review or not an intervention study). A total of 17 full-text studies were reviewed and ultimately 10 studies were included (Figure 1). All studies were RCTs, with the exception of a study by Mollard and Michaud [8], which was a nonrandomized controlled study.

### Study Results

A description of the quantitative evidence for the mHealth interventions reported in the included studies is presented in Table 1. In the 10 intervention studies, four different types of interventions were used: SMS reminders (for adherence to medication and physical activity plans) [26-28], web apps (for information and disease monitoring) [13,29-32], smartphone apps (for disease monitoring) [8], and pedometers (for activity tracking) [33]. Measured outcomes and study methods varied among the trials, depending on the aim of the mHealth intervention. All of the studies included between-group comparisons.

**Table 1:** Evidence for effectiveness of mobile health trials in patients with RA.

Ref^a^	Pts^b^ with RA^c^, n	Study duration (+ follow-up)	Intervention	Outcome measure	Results^d^		
					Intervention, mean (SD)	Control, mean (SD)	*P* value
[8]	63	6 mths^e^	LiveWith Arthritis smartphone app with optical imaging	Self-efficacy: P-SEMS^f^; PAM^g^	P-SEMS: 2.80^h^; PAM: 6.37^h^	P-SEMS: 1.66^h^; PAM: 2.30^h^	P-SEMS: .04; PAM: .46
[13]	160	12 mths	Physical activity intervention (web app)	Participants that meet Dutch public health physical activity recommendation (%)	6 mths^i^: 38; 12 mths^i^: 26	6 mths^i^: 22; 12 mths^i^: 15	.04; .12
[26]	96	6 mths	SMS reminders	CQR-9^j^	3.32 (5.66)	–0.14 (7.56)	.02
[27]	20	16 wks^k^	Motivational counselling and SMS reminders	Daily sitting time (hours/day)	–0.30 (1.90)	0.15 (1.43)	—^l^
[28]	150	16 wks	Motivational counselling and SMS reminders	Daily sitting time (hours/day)	–1.61 (CI –1.97 to –1.25)	0.59 (CI 0.24 to 0.95)	<.001
[29]	157	2 mths + 2 mths	Access to different sections of a web app: (1) social support, (2) gaming, (3) information, (4) 1 and 2, and (5) control group	Physical activity (β)^m^, (minutes); health care use (β), (number of visits); medication overuse (β), (POMI)^n,o^	Physical activity, group 4: β=3.39^p^; health care use, group 2: β=–0.41, group 4^p^: β=–0.33	N/A^q^	Physical activity, group 4: .02; health care use, group 2: .01, group 4: .02
[30]	320	12 mths	Sanoïa (web app)	Patient-physician interaction (PEPPI-5)^r^	0.6 (5.52)	–0.91 (6.08)	.01
[31]	44	12 mths	Telemonitoring with RETE-MARCHE (web app)	Patients with CDAI^s^ remission and comprehensive disease control after 1 year (%)	38.1	25	<.01
[32]	108	10 wks + 9 mths	Educational modules for improving self-efficacy in self-management of RA (web app)^t^	Self-efficacy (ASES)^u^	PI^v^: 83.9 (19.0); 9 mths PI: 84.1 (16.3)	PI: 68.5 (23.8); 9 mths PI: 68.6 (23.3)	PI: <.001; 9 mths PI: <.001
[33]	96	21 wks	Activity tracking with pedometer w^w^ or wo^x^ step targets	Physical activity (steps/day); Fatigue (PROMIS^y^-fatigue)	Steps/day w: 1656 (2161), wo: 1441 (2829); Fatigue w: –4.8 (7.7), wo: –3.2 (7.2)	Steps/day: –747 (3064); Fatigue: –1.6 (8.1)	Steps/day: .003; Fatigue: .21

^a^Ref: reference.

^b^Pts: patients.

^c^RA: rheumatoid arthritis.

^d^In the results column, between-group differences are presented.

^e^mths: months.

^f^P-SEMS: Patient-Reported Outcomes Measurement Information System Self-Efficacy Managing Symptoms.

^g^PAM: Patient Activation Measure.

^h^SD unknown.

^i^Intention-to-treat analysis.

^j^CQR-9: 9-item Compliance Questionnaire-Rheumatology.

^k^wks: weeks.

^l^—: not available.

^m^Unstandardized beta-coefficient (β) of multilevel linear model, including time exposed to intervention; no univariate results presented.

^n^POMI: Prescription Opioid Misuse Index.

^o^No significant differences were found in medication overuse.

^p^No significant differences in the other groups.

^q^N/A: not applicable.

^r^PEPPI-5: 5-item Perceived Efficacy in Patient-Physician Interactions.

^s^CDAI: Clinical Disease Activity Index.

^t^No primary outcome was defined.

^u^ASES: Arthritis Self-Efficacy Scale.

^v^PI: postintervention.

^w^w: with.

^x^wo: without.

^y^PROMIS: Patient-Reported Outcomes Measurement Information System.

### SMS Reminders

Three RCTs investigated the use of SMS reminders. Mary et al [26] evaluated the impact of weekly text messages on medication adherence in patients taking methotrexate for RA. Study patients who received reminder text messages on the day they had to take their methotrexate had a greater increase in medication adherence, as assessed using the 9-item Compliance Questionnaire-Rheumatology (CQR-9), compared with a control group and a group receiving a 15-minute pharmacist counselling session [26].

Two other RCTs [27,28] evaluated the effect of three individual motivational counselling sessions and individually tailored text message reminders on reducing daily sitting time. Initially, the pilot study [27] showed feasibility of the study design and acceptability of the intervention; a second study [28] evaluated the effect of the intervention on a larger population. In the intervention group of both studies, individually tailored text messages were sent to each participant to remind them of their behavioral goal(s). Participants could indicate their desired frequency of reminders (between 1 and 5 per week) [27,28]. Ultimately, patients in the intervention group reduced their daily sitting time by 1.61 hours per day [28].

### Web Apps

Five RCTs investigated the use of an online platform [13,29-32]. The online platforms were used as informative tools for patients and offered a way to self-monitor disease activity. The platforms demonstrated statistically significant effects in terms of self-management skills, patient empowerment, patient-physician interaction, and physical activity [13,29,30,32].

Allam et al [29] evaluated the effect of different sections of a web app on physical activity, health care utilization, and medication overuse. In the 4 intervention arms of the trial, patients received access to (1) the information section of the web app alone, (2) the information section combined with the social support section of the app, (3) the gaming section of the app, or (4) both the social support section and the gaming section. The intervention arms were compared with a control group that received no access to the web app. Patients that had access to the social support sections on the website decreased health care utilization and medication overuse, and patients with access to gamification features alone or combined with social support increased physical activity and decreased health care utilization [29].

Shigaki et al [32] evaluated the use of an online platform to improve self-efficacy, quality of life, health status, and pain. The platform combined individual and community features. Individual features included educational modules encouraging positive coping strategies for enhancing self-efficacy. In addition to online features, each member was provided with one-on-one leader support through weekly phone contact, typically lasting between 15 and 30 minutes. The platform improved self-efficacy and quality of life in the intervention group: no statistically significant improvements were seen in terms of health status or pain in the intervention group. Data collected through self-monitoring with patient-reported outcomes (PROs) were used for clinical decision making in one study [31]. In the study, PROs were remotely collected to evaluate disease activity by making use of a web app. A total of 44 patients were randomly allocated into 2 groups: the telemonitoring intensive strategy (TIS) group or the conventional strategy (control) group. In the TIS group, patients were monitored intensively and treated according to strict protocols. More patients in the TIS group achieved Clinical Disease Activity Index (CDAI) remission versus patients in the control group (38.1% versus 25% at 1 year; *P*<.01). Moreover, remission was achieved more rapidly, with a median of 20 weeks versus a median of over 36 weeks (*P*<.001)) [31].

### Smartphone App

A smartphone app was used in one study [8]. In the study, the use and feasibility of optical imaging through the smartphone were tested to monitor the progression of RA inflammation and deformity in patients’ hands. Inflammation and deformity were recorded by taking photos using the smartphone’s camera with a standardized procedure. The app also supported self-management behaviors with features to monitor symptoms and record lifestyle and environmental data (eg, diet, activity, and weather). After 6 months of app use, there was a statistically significant improvement in Patient-Reported Outcomes Measurement Information System (PROMIS) Self-Efficacy Managing Symptoms (P-SEMS) [8]. Results of the accuracy of the use of optical imaging for diagnosing flares of arthritis were not presented [8].

### Activity Tracking

A pedometer was used in one study [33], as a tool to monitor and improve physical activity. The overarching aim of the study was to reduce fatigue, measured with the 7-item PROMIS-fatigue questionnaire. Patients were randomly assigned into one of three parallel arms: (1) physical activity education only, (2) a pedometer plus step-monitoring diary, or (3) a pedometer that visualized step counts combined with goal setting. In both pedometer groups, the number of steps increased significantly, and changes differed significantly with the education-only group. Within-group changes in PROMIS-fatigue scores in both intervention groups were statistically significant (pedometer plus diary group, *P*=.02, and pedometer plus goal setting group, *P*<.001) [33]. However, the between-group difference in fatigue scores over time between the intervention and control groups were not statistically significant (*P*=.21) [33].

### Risk-of-Bias Assessment

**Figure 2:**
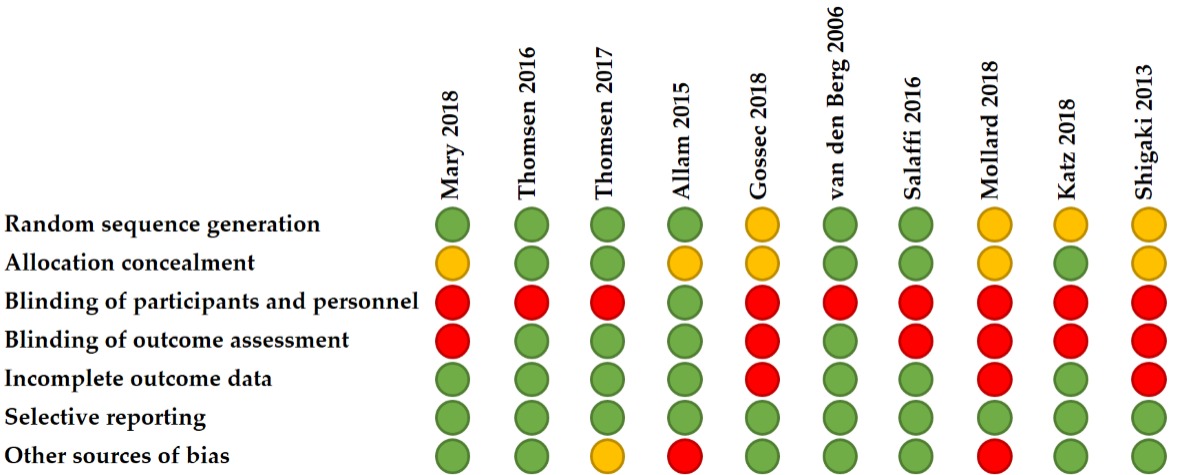
Assessment of risk of bias with the Cochrane Collaboration’s tool. Green=low risk of bias, red=high risk of bias, and orange=unclear risk of bias.

At least one domain of the risk-of-bias tool was scored as “at risk for bias in all studies.” Blinding participants was not performed in 9 out of 10 studies. No studies were classified as at high risk for selective reporting. The majority of studies (6/10, 60%) were at risk for bias on multiple domains, and in 70% (7/10) of studies, there was an unclear risk for bias on at least one domain. Results are presented in Figure 2.

## Discussion

### Summary

With this review, we provide an overview of the effects of mHealth interventions tested in clinical trials in patients with RA. We identified 10 studies that examined 4 different types of mHealth tools [8,13,26-33]. Web apps were the most tested mHealth intervention [13,29,30,32]. In 9 studies, significant desirable effects were reported on five main outcome measures: medication compliance (SMS reminders) [31], various domains of physical activity (pedometer, SMS reminders, and web apps) [13,27-29,33], percentage of patients that reach remission after 1 year (web app) [31], patient-physician interaction (web app) [30], and self-efficacy (smartphone and web apps) [8,32]. However, all studies were at risk for bias on at least one domain. Due to the heterogeneity in study outcomes and methods used, and the risk of bias in all studies, the promising effect of asynchronous mHealth on all the aforementioned outcomes needs to be corroborated in future studies.

### Principal Results

The results of the studies in this review show that it is possible with mHealth interventions to effectively monitor patients to achieve remission sooner [31]. Also, by sending reminders, mHealth tools can motivate patients to improve medication compliance [26], as well as to be more physically active [13,28,33]. Furthermore, mHealth can improve the self-efficacy of patients with RA [8,30,32]. If these results are corroborated, mHealth may contribute to better overall health [34,35], less health care usage, and possibly lower health care costs. In addition, we hypothesize that the value of patient-physician consultations that do happen can be increased, as several reviewed studies reported higher self-efficacy scores and better patient-physician communication. However, outcomes and study methods used in the mHealth studies were very heterogeneous, and studies were often at risk of methodological biases, which was also found in other mHealth reviews [15,36]. Heterogeneity was found in the wide range of interventions (smartphone apps, web apps, pedometers, SMS reminders) and chosen outcome measures. For instance, several different outcomes were measured (eg, self-efficacy, physical activity, and time to reach remission) and several different measures of self-efficacy were used (eg, Arthritis Self-Efficacy Scale, Patient Activation Measure, and P-SEMS) [8,32]. Furthermore, bias could have been introduced into most of the study results, as blinding of patients to their intervention was rarely achieved. Even though practical limitations often impede blinding in mHealth studies, it is recommended to ensure comparable experiences between control and intervention participants [37]. The study of Allam et al [29] illustrates that this can be achieved by utilizing a factorial study design; other options include an early-versus-delayed study design or partial blinding [37].

### Expectations

**Figure 3:**
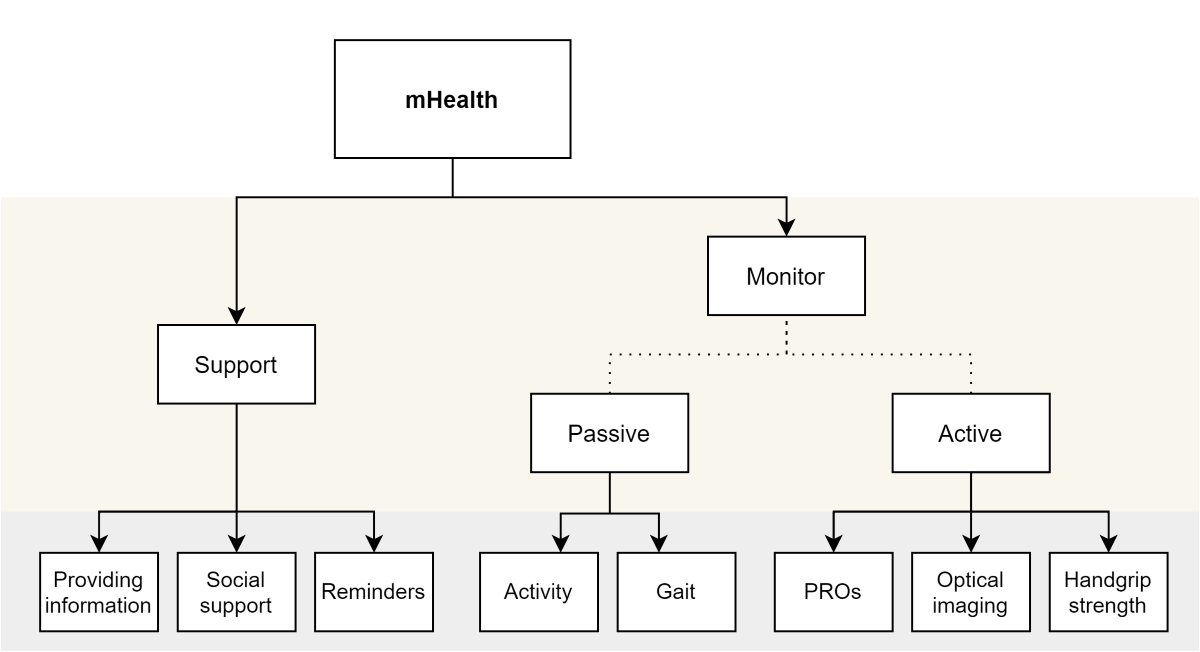
Identification of asynchronous mobile health (mHealth) uses in rheumatoid arthritis. PROs: patient-reported outcomes.

Based on the limited evidence available from mHealth intervention studies, we will cautiously discuss some potentially promising implementations and expectations. We distinguished two main implementations of asynchronous mHealth: (1) monitoring of patients, which can be subdivided into active and passive monitoring, and (2) supporting patients in their health behavior (Figure 3). With the help of remote monitoring through mHealth, unnecessary consultations might be prevented. In inflammatory bowel disease, a monitoring mHealth intervention has shown to reduce health care visits (33%) and hospital admissions, without increasing disease activity or decreasing patient satisfaction [19]. We expect that monitoring mHealth tools could also be very useful to patients with a stable, chronic rheumatic disease. Often, the value of a consultation is low, as 75% of patients with RA in routine clinical follow-up have low disease activity or are in remission [38]. With monitoring mHealth tools, these consultations could be avoided, which could in turn decrease health care costs. Fewer consultations, however, may also delay biological tapering or treatment when necessary. The safety and cost-effectiveness of this form of monitoring mHealth is currently investigated in two RCTs [20,39]. We anticipate that the use of monitoring mHealth interventions (telemonitoring) will increase based on the increasing number of studies performed with mHealth and the increasing use of smartphones in the population. For patients with RA and their health care providers, objective parameters of disease activity that do not require patient effort (ie, filling in a questionnaire) but are automatically collected would be ideal. That is because passive, remote monitoring eliminates the need for active sharing of disease outcomes and would therefore (partly) surpass adherence issues. Ultimately, this would lead to less missing data and lower the burden for patients. Devices can already collect and share these data without any participation of the patient, and uses in rheumatology are being explored [10,11,40,41] (Figure 3). mHealth that supports patients in their health behavior (supporting mHealth) can help patients adhere better to healthy lifestyles and medication, and can play a significant role in helping patients to cope with their disease by means of social support, education, and improving self-efficacy [13,26-30,32]. In this review, studies used supportive mHealth to get patients more physically active, improve their self-efficacy, and increase their medication adherence [13,26-30,32]. In other medical fields, similar results were found with positive effects in terms of lifestyle and medication adherence [42-44]. This indicates that supportive mHealth may play an important role in preventive (health behavior change) medicine. However, one important gap in our knowledge here is that it is unclear how long the effects of these interventions last. No study in this review had a follow-up of longer than 1 year, and effects often decreased over time. This is likely due to the decrease in adherence to the intervention, which impedes the long-term impact. To increase adherence, some studies have reported on the use of persuasive elements in mHealth tools, such as the use of gamification or persuasive principles in text reminders [29,45]. We expect this to be an important line of research to increase adherence to mHealth tools and to ultimately optimize the impact of mHealth interventions.

### Strengths and Limitations

This review was broad in scope and did not focus on one type of mHealth intervention, allowing it to provide a clear overview of the current position of all mHealth tools tested in clinical trials in patients with RA. To maintain a broad scope, the study regarded web apps as mHealth tools because web apps are easily accessible through mobile phones. However, it is possible, that the adoption of, or adherence to, web apps is different on computers and mobile phones. This might lead to other outcomes of the interventions, which should be further examined in future research. Lastly, it is possible that due to biased preference, only studies with positive results are published, which could have led to an overrepresentation of positive effects in this review. However, there is little evidence that this occurred, as we examined trial registries (clinicaltrials.gov and isrctn.com) and encountered only one trial that was completed more than 2 years ago, which had not published its results.

### Conclusion

There is a limited number of studies assessing the effect of asynchronous mHealth interventions in patients with RA. The available studies show that asynchronous mHealth interventions have been used to monitor patients or to support them in their health behavior. The reviewed studies reported significant beneficial results of SMS reminders, web apps, and smartphone apps on several outcomes. However, study methods varied widely, all studies were at risk of bias, and follow-up length was often short. Therefore, the results of the review indicate that all reviewed types of mHealth interventions show some promise, but also that these results need to be corroborated in future studies.

## Appendix

Multimedia Appendix 1 Search strategy.

## Abbreviations

CDAI: Clinical Disease Activity Index
CQR-9: 9-item Compliance Questionnaire-Rheumatology
mHealth: mobile health
PRISMA: Preferred Reporting Items for Systematic Reviews and Meta-Analysis
PROs: patient-reported outcomes
PROMIS: Patient-Reported Outcomes Measurement Information System
P-SEMS: Patient-Reported Outcomes Measurement Information System Self-Efficacy Managing Symptoms
RA: rheumatoid arthritis
RCT: randomized controlled trial
TIS: telemonitoring intensive strategy
