# Supplementary material for: Asynchronous mHealth Interventions in Rheumatoid Arthritis: Systematic Scoping Review
Source: JMIR Mhealth Uhealth. 2020 Nov 5;8(11):e19260. doi: 10.2196/19260 (PMC7677027; doi:10.2196/19260)
Supplement: Multimedia Appendix 1 [file mhealth_v8i11e19260_app1.docx]

For the Supplementary Information

**PubMed Session Results (01 Nov 2019)**

| **#** | **Query** | **Results** |
| --- | --- | --- |
| #5 | #3 OR #4 | 286 |
| #4 | telerheumatology[tiab] OR tele-rheumatology[tiab] OR e-rheumatology[tiab] | 12 |
| #3 | #1 AND #2 | 285 |
| #2 | "Rheumatology"[Mesh] OR "Rheumatic Diseases"[Mesh:noexp] OR "Arthritis, Rheumatoid"[Mesh:noexp] OR rheumatoid arthriti*[tiab] OR rheumatic arthritis[tiab] OR rheumatic polyarthritis[tiab] OR rheumatoid polyarthritis[tiab] OR articular rheumatism[tiab] OR chronic progressive polyarthritis[tiab] | 153,335 |
| #1 | "Telemedicine"[Mesh] OR "Telenursing"[Mesh] OR "User-Computer Interface"[Mesh] OR "Mobile Applications"[Mesh] OR "Cell phone"[Mesh] OR "Cell Phone Use"[Mesh] OR "Computers, Handheld"[Mesh] OR "Wearable Electronic Devices"[Mesh] OR ehealth*[tiab] OR e-health*[tiab] OR mhealth*[tiab] OR m-health*[tiab] OR mobile health*[tiab] OR digital health[tiab] OR app[tiab] OR apps[tiab] OR smartphone*[tiab] OR smart phone*[tiab] OR smartwatch*[tiab] OR smart watch*[tiab] OR phone application*[tiab] OR telephone application*[tiab] OR watch application*[tiab] OR mobile application*[tiab] OR mobile technolog*[tiab] OR mobile device*[tiab] OR smart device*[tiab] OR health application*[tiab] OR ipad[tiab] OR ipads[tiab] OR iphone*[tiab] OR android[tiab] OR whatsapp*[tiab] OR wearable*[tiab] OR facebook[tiab] OR cell phone*[tiab] OR cellular phone*[tiab] OR mobile phone*[tiab] OR game*[tiab] OR gaming[tiab] OR gamification[tiab] OR computer tablet*[tiab] OR tablet computer*[tiab] OR tablet device*[tiab] OR electronic device*[tiab] OR telemonitor*[tiab] OR tele monitor*[tiab] | 200,430 |

**Embase.com Session Results (01 Nov 2019)**

| **#** | **Query** | **Results** |
| --- | --- | --- |
| #5 | #3 OR #4 | 1,088 |
| #4 | telerheumatology:ab,ti,kw OR 'tele-rheumatology':ab,ti,kw OR 'e-rheumatology':ab,ti,kw | 26 |
| #3 | #1 AND #2 | 1,078 |
| #2 | 'rheumatology'/exp OR 'rheumatic disease'/de OR 'rheumatoid arthritis'/de OR 'rheumatoid arthriti*':ab,ti,kw OR 'rheumatic arthritis':ab,ti,kw OR 'rheumatic polyarthritis':ab,ti,kw OR 'rheumatoid polyarthritis':ab,ti,kw OR 'articular rheumatism':ab,ti,kw OR 'chronic progressive polyarthritis':ab,ti,kw | 281,881 |
| #1 | 'telehealth'/exp OR 'computer interface'/exp OR 'mobile application'/exp OR 'mobile phone'/exp OR 'cell phone use'/exp OR 'personal digital assistant'/exp OR ehealth*:ab,ti,kw OR 'e-health*':ab,ti,kw OR mhealth*:ab,ti,kw OR 'm-health*':ab,ti,kw OR 'mobile health*':ab,ti,kw OR 'digital health':ab,ti,kw OR app:ab,ti,kw OR apps:ab,ti,kw OR smartphone*:ab,ti,kw OR 'smart phone*':ab,ti,kw OR smartwatch*:ab,ti,kw OR 'smart watch*':ab,ti,kw OR 'phone application*':ab,ti,kw OR 'telephone application*':ab,ti,kw OR 'watch application*':ab,ti,kw OR 'mobile application*':ab,ti,kw OR 'mobile technolog*':ab,ti,kw OR 'mobile device*':ab,ti,kw OR 'smart device*':ab,ti,kw OR 'health application*':ab,ti,kw OR ipad:ab,ti,kw OR ipads:ab,ti,kw OR iphone*:ab,ti,kw OR android:ab,ti,kw OR whatsapp*:ab,ti,kw OR wearable*:ab,ti,kw OR facebook:ab,ti,kw OR 'cell phone*':ab,ti,kw OR 'cellular phone*':ab,ti,kw OR 'mobile phone*':ab,ti,kw OR game*:ab,ti,kw OR gaming:ab,ti,kw OR gamification:ab,ti,kw OR 'computer tablet*':ab,ti,kw OR 'tablet computer*':ab,ti,kw OR 'tablet device*':ab,ti,kw OR 'electronic device*':ab,ti,kw OR telemonitor*:ab,ti,kw OR 'tele monitor*':ab,ti,kw | 232,991 |

**Ebsco / PsycINFO Session Results (01 Nov 2019)**

| **#** | **Query** | **Results** |
| --- | --- | --- |
| S5 | S3 OR S4 | 9 |
| S4 | TI (telerheumatology OR "tele-rheumatology" OR "e-rheumatology") OR AB (telerheumatology OR "tele-rheumatology" OR "e-rheumatology") OR KW (telerheumatology OR "tele-rheumatology" OR "e-rheumatology") | 0 |
| S3 | S1 AND S2 | 9 |
| S2 | DE "Rheumatoid Arthritis" OR TI ("rheumatoid arthriti*" OR "rheumatic arthritis" OR "rheumatic polyarthritis" OR "rheumatoid polyarthritis" OR "articular rheumatism" OR "chronic progressive polyarthritis") OR AB ("rheumatoid arthriti*" OR "rheumatic arthritis" OR "rheumatic polyarthritis" OR "rheumatoid polyarthritis" OR "articular rheumatism" OR "chronic progressive polyarthritis") OR KW ("rheumatoid arthriti*" OR "rheumatic arthritis" OR "rheumatic polyarthritis" OR "rheumatoid polyarthritis" OR "articular rheumatism" OR "chronic progressive polyarthritis") | 2,759 |
| S1 | DE "Telemedicine" OR DE "Human Computer Interaction" OR DE "Mobile Devices" OR DE "Cellular Phones" OR TI (ehealth* OR "e-health*" OR mhealth* OR "m-health*" OR "mobile health*" OR "digital health" OR app OR apps OR smartphone* OR "smart phone*" OR smartwatch* OR "smart watch*" OR "phone application*" OR "telephone application*" OR "watch application*" OR "mobile application*" OR "mobile technolog*" OR "mobile device*" OR "smart device*" OR "health application*" OR ipad OR ipads OR iphone* OR android OR whatsapp* OR wearable* OR facebook OR "cell phone*" OR "cellular phone*" OR "mobile phone*" OR game* OR gaming OR gamification OR "computer tablet*" OR "tablet computer*" OR "tablet device*" OR "electronic device*" OR telemonitor* OR "tele monitor*") OR AB (ehealth* OR "e-health*" OR mhealth* OR "m-health*" OR "mobile health*" OR "digital health" OR app OR apps OR smartphone* OR "smart phone*" OR smartwatch* OR "smart watch*" OR "phone application*" OR "telephone application*" OR "watch application*" OR "mobile application*" OR "mobile technolog*" OR "mobile device*" OR "smart device*" OR "health application*" OR ipad OR ipads OR iphone* OR android OR whatsapp* OR wearable* OR facebook OR "cell phone*" OR "cellular phone*" OR "mobile phone*" OR game* OR gaming OR gamification OR "computer tablet*" OR "tablet computer*" OR "tablet device*" OR "electronic device*" OR telemonitor* OR "tele monitor*") OR KW (ehealth* OR "e-health*" OR mhealth* OR "m-health*" OR "mobile health*" OR "digital health" OR app OR apps OR smartphone* OR "smart phone*" OR smartwatch* OR "smart watch*" OR "phone application*" OR "telephone application*" OR "watch application*" OR "mobile application*" OR "mobile technolog*" OR "mobile device*" OR "smart device*" OR "health application*" OR ipad OR ipads OR iphone* OR android OR whatsapp* OR wearable* OR facebook OR "cell phone*" OR "cellular phone*" OR "mobile phone*" OR game* OR gaming OR gamification OR "computer tablet*" OR "tablet computer*" OR "tablet device*" OR "electronic device*" OR telemonitor* OR "tele monitor*") | 81,995 |

**Wiley / Cochrane Library Session Results (01 Nov 2019)**

| **#** | **Query** | **Results** |
| --- | --- | --- |
| #5 | #3 OR #4 | 81 |
| #4 | (telerheumatology OR (tele NEXT rheumatology) OR (e NEXT rheumatology)):ab,ti,kw | 1 |
| #3 | #1 AND #2 | 80 |
| #2 | ((rheumatoid NEXT arthriti*) OR "rheumatic arthritis" OR "rheumatic polyarthritis" OR "rheumatoid polyarthritis" OR "articular rheumatism" OR "chronic progressive polyarthritis"):ab,ti,kw | 14,569 |
| #1 | (ehealth* OR (e NEXT health*) OR mhealth* OR (m NEXT health*) OR (mobile NEXT health*) OR "digital health" OR app OR apps OR smartphone* OR (smart NEXT phone*) OR smartwatch* OR (smart NEXT watch*) OR (phone NEXT application*) OR (telephone NEXT application*) OR (watch NEXT application*) OR (mobile NEXT application*) OR (mobile NEXT technolog*) OR (mobile NEXT device*) OR (smart NEXT device*) OR (health NEXT application*) OR ipad OR ipads OR iphone* OR android OR whatsapp* OR wearable* OR facebook OR (cell NEXT phone*) OR (cellular NEXT phone*) OR (mobile NEXT phone*) OR game* OR gaming OR gamification OR (computer NEXT tablet*) OR (tablet NEXT computer*) OR (tablet NEXT device*) OR (electronic NEXT device*) OR telemonitor* OR (tele NEXT monitor*)):ab,ti,kw | 17,382 |

**Scopus Session Results (01 Nov 2019)**

| **#** | **Query** | **Results** |
| --- | --- | --- |
| #5 | #3 OR #4 | 288 |
| #4 | TITLE-ABS-KEY ( telerheumatology OR {tele-rheumatology} OR {e-rheumatology} ) | 14 |
| #3 | #1 AND #2 | 276 |
| #2 | TITLE-ABS-KEY ( "rheumatoid arthriti*" OR {rheumatic arthritis} OR {rheumatic polyarthritis} OR {rheumatoid polyarthritis} OR {articular rheumatism} OR {chronic progressive polyarthritis} ) | 187,400 |
| #1 | TITLE-ABS-KEY ( ehealth* OR "e-health*" OR mhealth* OR "m-health*" OR "mobile health*" OR {digital health} OR app OR apps OR smartphone* OR "smart phone*" OR smartwatch* OR "smart watch*" OR "phone application*" OR "telephone application*" OR "watch application*" OR "mobile application*" OR "mobile technolog*" OR "mobile device*" OR "smart device*" OR "health application*" OR ipad OR ipads OR iphone* OR android OR whatsapp* OR wearable* OR facebook OR "cell phone*" OR "cellular phone*" OR "mobile phone*" OR game* OR gaming OR gamification OR "computer tablet*" OR "tablet computer*" OR "tablet device*" OR "electronic device*" OR telemonitor* OR "tele monitor*" ) | 702,516 |
